# TLR-2 mediated cytosolic-Ca^2+^ surge activates ER-stress-superoxide-NO signalosome augmenting TNF-α production leading to apoptosis of *Mycobacterium smegmatis*-infected fish macrophages

**DOI:** 10.1038/s41598-019-48847-1

**Published:** 2019-08-23

**Authors:** Md. Arafat Hussain, Debika Datta, Rashmi Singh, Manmohan Kumar, Jai Kumar, Shibnath Mazumder

**Affiliations:** 1 Immunobiology Laboratory, Department of Zoology, University of Delhi, Delhi, 110007 India; 2 Faculty of Life Sciences & Bio-Technology, South Asian University, New Delhi, 110021 India

**Keywords:** Immune cell death, Innate immune cells

## Abstract

The implications of TLR-2 mediated alterations in cytosolic-Ca^2+^((Ca^2+^)_c_) levels in *M*. *smegmatis* infections is not well known. Using headkidney macrophages (HKM) from *Clarias gariepinus*, we observed TLR-2 signalling is required in the phagocytosis of *M*. *smegmatis*. *M*. *smegmatis* induced caspase-dependent HKM apoptosis in MOI, time and growth-phase dependent manner. RNAi and inhibitor studies demonstrated critical role of TLR-2 in eliciting (Ca^2+^)_c_-surge and c-Src-PI3K-PLC axis playing an intermediary role in the process. The (Ca^2+^)_c_-surge triggered downstream ER-stress and superoxide (O_2_^−^) generation. The cross-talk between ER-stress and O_2_^−^ amplified TNF-α production, which led to HKM apoptosis and bacterial clearance. Release of nitric oxide (NO) was also observed and silencing the NOS2-NO axis enhanced intracellular bacterial survival and attenuated caspase activity. Pre-treatment with diphenyleneidonium chloride inhibited NO production implicating O_2_^−–^NO axis imperative in *M*. *smegmatis-*induced HKM apoptosis. NO positively impacted CHOP expression and TNF-α production in infected HKM. We conclude that, TLR-2 induced (Ca^2+^)_c_-surge and ensuing cross-talk between ER-stress and O_2_^−^ potentiates HKM pathology by amplifying pro-inflammatory TNF-α production. Moreover, the pro-oxidant environment triggers NO release which prolonged ER-stress and TNF-α production, culminating in HKM apoptosis and bacterial clearance. Together, our study suggests HKM an alternate model to study macrophage-mycobacteria interactions.

## Introduction

*M*. *smegmatis*, rapidly growing atypical mycobacteria is classified with other rapid growers *M*. *fortuitum*, *M*. *abscessus*, and *M*. *chelonei* into Runyon group IV of mycobacteria^1^ Due to its fast growing nature, *M*. *smegmatis* is used to understand mycobacterial physiology. It also serves as fast, safe surrogate for screening of potential drugs^2,3^ and studying the development of drug resistance in mycobacteria^4^.

*M*. *smegmatis* though considered saprophytic, incidences of animal infections in natural and laboratory conditions has been reported^5,6^, therefore a suitable model is warranted to study the bacterium. The pathogenic potential of *M*. *smegmatis* in human was first recognized by Vonmoos *et al*.^7^, and since then, reports implicating its role in cellulitis, soft tissue necrosis, localized abscesses, osteomyelitis, lipoid pneumonia and healthcare-associated diseases are increasing^8,9^. Advent of drug-resistant strains^10^ coupled with reports of the bacterium from immunocompromised individuals^11^ requires revisiting *M*. *smegmatis* pathogenesis.

Toll like receptor-2 (TLR-2) plays important role in the recognition and phagocytosis of mycobacteria^12^. Earlier studies suggested TLR-2 signalling induces apoptosis in mycobacteria-infected macrophages^13^. Apoptosis in mycobacterial infections is a matter of conjecture; some studies correlate apoptosis with clearance of bacteria from infected cells while others document that virulent mycobacteria utilizes the apoptotic machinery for spread and persistence^14^. The alterations in (Ca^2+^)_c_ homoeostasis of mycobacteria-infected macrophages has been implicated both as pro- and anti-apoptotic in different studies^15,16^. TLR-2 induces release of intracellular Ca^2+^ through IP3 receptors^17^ but the same has not been yet reported in *M*. *smegmatis* infection. Once released, Ca^2+^ either binds to various Ca^2+^-binding proteins or enters the ER^18^. The influx of Ca^2+^ causes ER-stress leading to apoptosis in mycobacteria-infected cells^15^. It has been reported recently that *M*. *smegmatis* infection also induces ER-stress^19^ but TLR-2 involvement in the process is not known. Macrophages respond to mycobacterial infection by production of reactive oxygen species (ROS) which impact the outcome of infection^20^. The production of TLR-2 dependent ROS has been reported in tuberculin stimulated macrophages^21^. Although, ER-stress and ROS production has been observed in mycobacterial pathogenesis, the cross-talk between the two molecular events has not been reported.

The production of pro-inflammatory cytokine TNF-α leading to apoptosis of host macrophages has been reported in *M*. *smegmatis*-infected murine macrophages^19,22^. The apical role of TLR-2 in TNF-α production has been observed in *M*. *tuberculosis* infections^23^, however, it is yet to be reported against *M*. *smegmatis*. Macrophages produce NO in response to mycobacterial infection that inhibits bacterial growth and helps in clearance of the pathogen^24^. However, the role of NO in case of atypical mycobacterial pathogenesis is inconclusive^25^. We earlier reported the involvement of NO in apoptosis of *M*. *fortuitum*-infected macrophages *via* activation of caspase-8^15^. Interaction of O_2_^−^ with NO forms microbicidal peroxynitrite (ONOO^-^) intermediate^26^ but the association has not been well documented in *M*. *smegmatis* infection.

The fast growth rate, less stringent bio-safety requirements coupled with close homology with many of the *M*. *tuberculosis* virulence genes suggests *M*. *smegmatis* to be an appropriate model to study mechanisms underlying mycobacterial pathogenesis^27^ and in identifying novel intervention strategies^28^. Therefore, we aimed to develop an alternate *in vitro* model for *M*. *smegmatis* infection that can be used to understand mycobacterial pathogenesis.

Fish have well defined immune system comprising of both innate and adaptive components. It is strikingly similar and includes most if not all the elements of immune system in mammals^29^. Fish have been successfully used as model to understand molecular-pathogenesis and immunology of several diseases including tuberculosis^30^. Headkidney is a major immunocompetent organ in fish and HKM serve as the first line of defense^31^. Hitherto, the reports on the pathogenesis of *M*. *smegmatis* have been majorly on mammalian systems. We developed an infection model for *M*. *smegmatis* using HKM from *Clarias gariepinus*. This fish is available round the year, can adapt to laboratory conditions and have easily identifiable immune organs. Our earlier studies have indeed established that *C*. *gariepinius*-HKM are inherently phagocytic and serve as an excellent model for studying the pathogenesis of *M*. *fortuitum* and *A*. *hydrophila*^15,32^.

In this study we have used HKM to elucidate the apical role of TLR-2 during *M*. *smegmatis* infection. We demonstrate how TLR-2 induced (Ca^2+^)_c_-surge precedents ER-stress and O_2_^−^ generation triggering production of pro-apoptotic TNF-α and NO.

## Results

### Phagocytosis of live *M*. *smegmatis* induces HKM cytotoxicity

**Figure 1:**
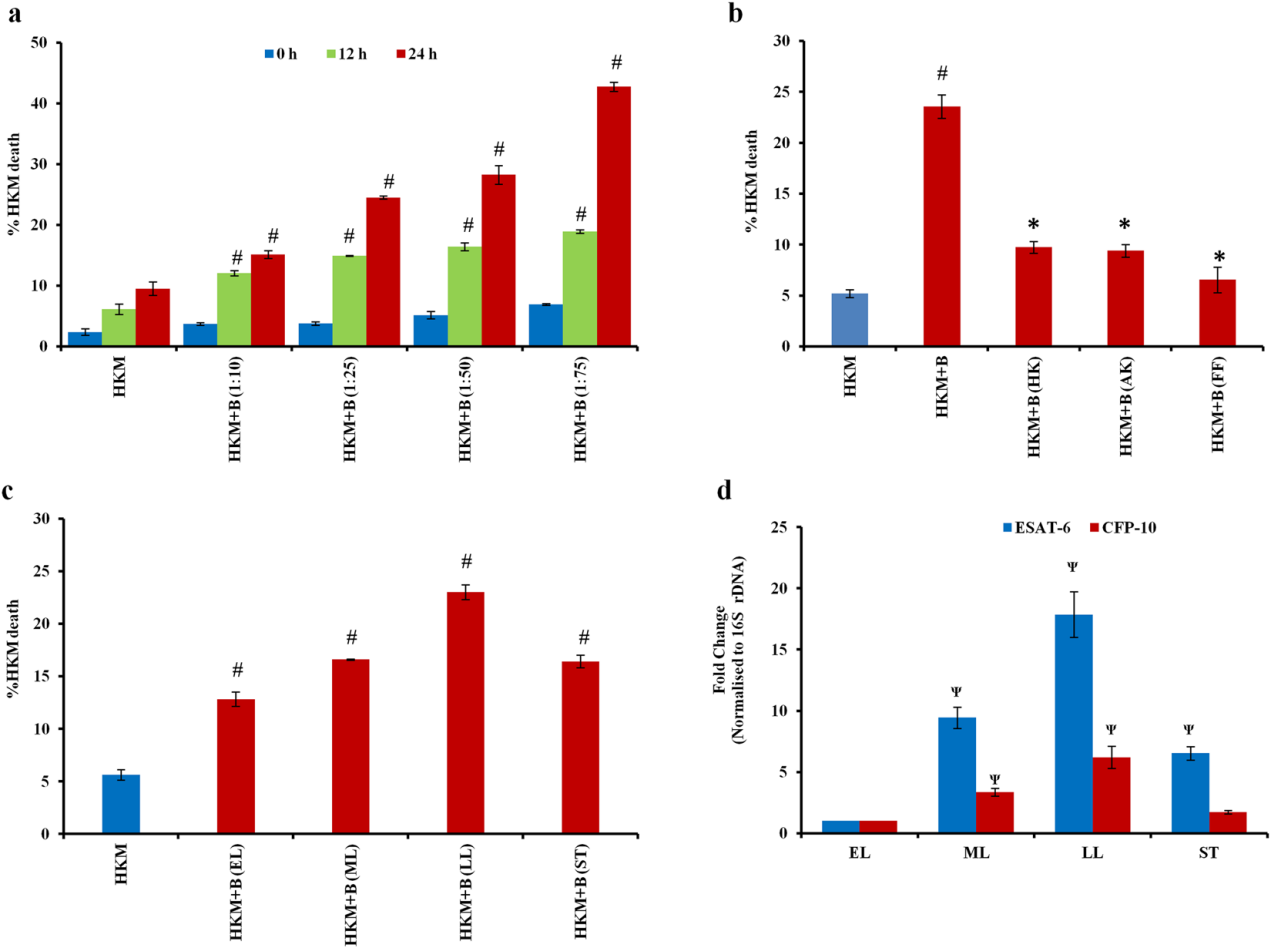
Live *M*. *smegmatis-*induced HKM death is MOI and growth-phase dependent. (**a**) HKM were infected with *M*. *smegmatis* at different MOI and percentage death measured at indicated time intervals p.i. (**b**) HKM were infected with *M*. *smegmatis* killed by different regimens and percentage death measured at 24 h p.i. (**c**) HKM were infected with *M*. *smegmatis* (MOI 1:25) harvested at indicated growth phases and percentage death measured at 24 h p.i. (**d**) Fold changes of ESAT-6 and CFP-10-mRNA expression in different phases of *M*. *smegmatis* growth. Vertical bars represent mean ± S.E.M (n = 3). ^#^*P* < 0.05 compared to HKM; ^*^P < 0.05 compared to HKM + B; ^Ψ^*P* < 0.05 compared to EL. HKM, uninfected HKM; HKM + B, HKM infected with live *M*. *smegmatis*; HKM + B (HK); HKM + B (AK), HKM + B (FF) HKM infected with heat killed, amikacin treated, formalin fixed *M*. *smegmatis* respectively; HKM + B (EL), HKM + B (ML), HKM + B (LL), HKM + B (ST), HKM infected with *M*. *smegmatis* harvested from early-log (EL), mid-log (ML), late-log (LL) and stationary phase (ST) respectively.

At the outset, we studied the effect of *M*. *smegmatis* infection on HKM. The cells were infected with range of MOI and viability checked by trypan blue dye exclusion method at indicated times p.i. We observed *M*. *smegmatis*-induced HKM death to be time and MOI dependent, with maximum cytotoxicity at 24 h p.i. (Fig. 1a). We selected MOI of 1: 25 (HKM: bacteria) as standard dose for further studies.

We were interested to know whether viability of *M*. *smegmatis* was important for inducing HKM cytotoxicity. HKM were treated with live and dead *M*. *smegmatis* at MOI of 1:25 and cell death studied 24 h p.i. by trypan blue. Several methods were adopted (heat-killed, amikacin treatment and formalin fixation) to kill the bacteria and each induced 100% bacterial death (data not shown). We observed HKM cytotoxicity was significantly attenuated on treatment with dead bacteria obtained from different treatment regimens (Fig. 1b) clearly suggesting that metabolically active *M*. *smegmatis* are proficient in inducing cytotoxicity.

Next, HKM were infected with *M*. *smegmatis* harvested from different phases of growth and cell death studied at 24 h p.i. It was observed that bacteria collected from late-log (LL) phase induced maximum HKM death followed by mid-log (ML), stationary (ST) and early-log (EL) phase respectively (Fig. 1c). Expression of virulence factors governs the pathogenicity of bacteria. To validate our findings, we monitored the expression of two virulent factors, early secretory antigenic target- 6 (ESAT-6) and culture filtrate protein (CFP-10) at different phases of *M*. *smegmatis* growth. Primers were designed (Supplementary Table 1) and real-time PCR results suggested the expression of ESAT-6 and CFP-10-mRNA *in sync* with the cytotoxic effects; highest expression recorded in late-log phase bacteria followed by mid-log, stationary and early-log phase respectively (Fig. 1d).

### *M*. *smegmatis*-induced HKM death is apoptotic

**Figure 2:**
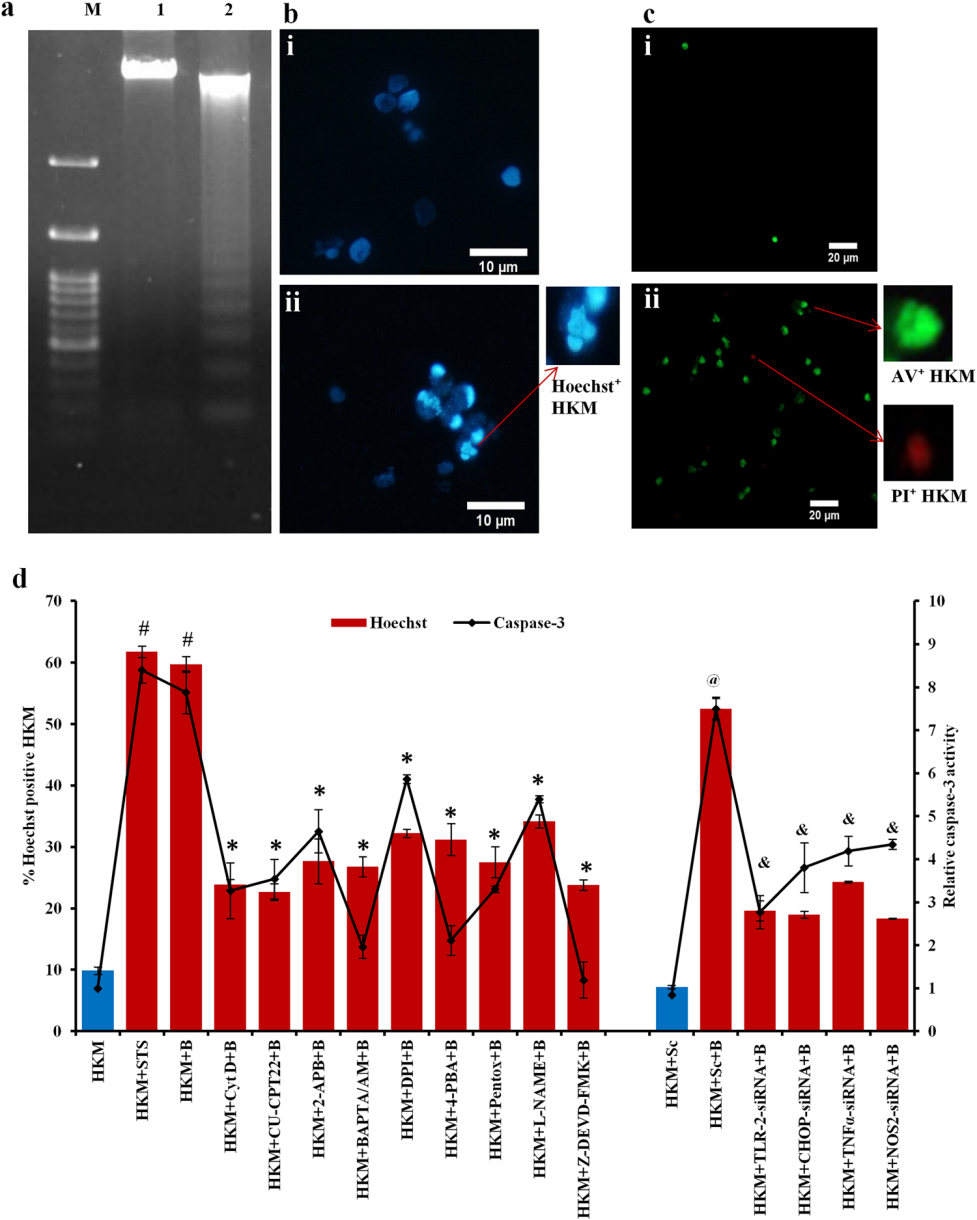
*M*. *smegmatis-*induced HKM death is apoptotic. (**a**) HKM were lysed at 24 h p.i, DNA isolated and subjected to agarose gel electrophoresis (1.2%) alongside 100 bp DNA marker. Lane M, marker; lane 1, DNA from uninfected HKM; lane 2, DNA from *M*. *smegmatis* infected HKM. (**b**) Uninfected and *M*. *smegmatis* infected HKM were stained with Hoechst 33342 at 24 h p.i. and observed under fluorescence microscope (×100), bi, bii represents uninfected and *M*. *smegmatis* infected HKM respectively. (**c**) Uninfected and infected HKM were stained with Annexin V-FITC propidium iodide at 24 h p.i. and observed under fluorescence microscope (×40) ci, cii represents uninfected and *M*. *smegmatis* infected HKM respectively. (**d**) HKM were pre-treated with or without specific inhibitors separately, transfected with sc-siRNA or specific siRNAs and apoptosis studied by enumerating Hoechst 33342 positive HKM and measuring relative caspase-3 activity at 24 h p.i. Vertical bars represent mean ± S.E.M (n = 3). ^#^*P* < 0.05 compared to HKM; *P < 0.05 compared to HKM + B; ^@^*P* < 0.05 compared to HKM + Sc; ^&^*P* < 0.05 compared to HKM + Sc + B. HKM^,^ uninfected HKM; HKM + STS, HKM treated with STS; HKM + B, HKM infected with *M*. *smegmatis*; HKM + CytD + B, HKM + CU-CPT22 + B, HKM + 2-APB + B, HKM + BAPTA/AM + B, HKM + DPI + B, HKM + 4-PBA + B, HKM + Pentox + B, HKM + L-NAME + B, HKM + Z-DEVD-FMK + B, HKM pre-treated with Cyt D, CU-CPT22, 2-APB, BAPTA/AM, DPI, 4-PBA, Pentox, L-NAME, Z-DEVD-FMK respectively and infected with *M*. *smegmatis*; HKM + Sc, HKM transfected with sc-siRNA; HKM + Sc + B, HKM + TLR-2-siRNA + B, HKM + CHOP-siRNA + B, HKM + TNF-α-siRNA + B, HKM + NOS2-siRNA + B, HKM transfected with sc-siRNA, TLR-2-siRNA, CHOP-siRNA, TNF-α-siRNA, NOS2-siRNA respectively and infected with *M*. *smegmatis*.

The next step was determining the nature of cell death induced by *M*. *smegmatis*. The HKM were infected at MOI of 1: 25 and we observed prominent oligonucleosomal DNA ladder, hallmark of apoptosis, in *M*. *smegmatis*-infected HKM (Fig. 2a). To further substantiate our finding, control and infected HKM were stained with Hoechst 33342 and AV-PI double stain respectively and at 24 h p.i. observed under fluorescence microscope. We noted significant intensely stained or Hoechst 33342 positive cells in infected HKM compared to uninfected HKM (Fig. 2bii). AV binds to exposed phosphatidyl serine on plasma-membrane of apoptotic cells while PI stains necrotic cells. In concordance with this, we observed significant AV positive (44.03 ± 5.4% P < 0.05) and few PI positive (7.91 ± 2.1%) *M*. *smegmatis*-infected HKM (Fig. 2cii). Few control HKM were AV-positive (4.61 ± 0.70%). These results established *M*. *smegmatis*-induced HKM death to be apoptotic in nature.

To check whether apoptosis induced by *M*. *smegmatis* is caspase-dependent, we monitored the activity of executioner caspase or caspase-3 and noted significant increase in caspase-3 activity in *M*. *smegmatis*-infected HKM. Pre-treatment with caspase-3 specific inhibitor Z-DEVD-FMK significantly reduced caspase-3 activity corroborating with the reduction in HKM apoptosis (Fig. 2d) suggesting *M*. *smegmatis*-induced HKM apoptosis to be caspase mediated. STS was used as positive control in the study.

### TLR-2 promotes phagocytosis of *M*. *smegmatis*

**Figure 3:**
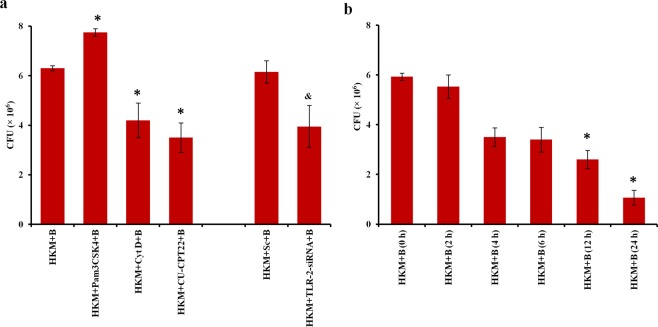
TLR-2 helps in innate recognition of *M*. *smegmatis*. (**a**) HKM pre-treated with indicated inhibitors or transfected with TLR-2-siRNA were infected with *M*. *smegmatis*, lysed after amikacin treatment and the viability of intracellular bacteria enumerated by MTT assay. (**b**) HKM were infected with *M*. *smegmatis* and viability of intracellular bacteria enumerated at indicated time points by MTT assay. Vertical bars represent mean ± S.E.M (n = 3). ^*^P < 0.05 compared to HKM + B and HKM + B (0 h); ^&^*P* < 0.05 compared to HKM + Sc + B. HKM + B, HKM infected with *M*. *smegmatis*; HKM + Pam3CSK4 + B, HKM + CytD + B, HKM + CU-CPT22 + B, HKM pre-treated with Pam3CSK4, Cyt D, CU-CPT22 respectively and infected with *M*. *smegmatis*; HKM + Sc + B, HKM + TLR-2-siRNA + B, HKM transfected with sc-siRNA, TLR-2-siRNA respectively and infected with *M*. *smegmatis*.

The importance of intracellular bacteria for apoptosis was studied by infecting HKM with *M*. *smegmatis* in the presence or absence of phagocytosis inhibitor, Cyt D. We observed Cyt D significantly interfered with phagocytosis of *M*. *smegmatis* (Fig. 3a) besides attenuating HKM apoptosis (Fig. 2d). Importantly, Cyt D failed to inhibit staurosporine-induced macrophage apoptosis (data not shown), clearly indicating that *M*. *smegmatis*-induced HKM apoptosis resulted from the phagocytosis of live bacteria.

The next step was studying the underlying mechanism of phagocytosis. HKM were infected with *M*. *smegmatis*, lysed at indicated time intervals and intracellular bacteria enumerated. We observed that HKM efficiently phagocytosed *M*. *smegmatis* and triggered steady decline in number of intracellular bacteria (Fig. 3b). Role of TLR-2 as PRR for different mycobacteria being well known^23^ was the prime target. HKM were infected with *M*. *smegmatis* and the expression of TLR-2 and its adaptor MyD88 monitored by real-time PCR. We observed significant upregulation in expression of both TLR-2 and MyD88 with maximum increase recorded at 30 min p.i. (Supplementary Fig. 1) implicating the role of TLR-2-MyD88 pathway in *M*. *smegmatis* pathogenesis. To further understand the role of TLR-2 in *M*. *smegmatis* infection, HKM were pre-treated with TLR-2 inhibitor CU-CPT22 or transfected with TLR-2-siRNA, then infected with *M*. *smegmatis*, lysed at 0 h p.i. (4 h internalisation + 1 h amikacin treatment) and bacteria enumerated. Significant reduction in intracellular bacterial load suggested the role of TLR-2 in phagocytosis of *M*. *smegmatis* by HKM. Pam3CSK4 was used as positive control in the study which enhanced *M*. *smegmatis* phagocytosis (Fig. 3a). To this we concluded that TLR-2 signalling helps in phagocytosis of *M*. *smegmatis*.

### TLR-2-induced elevation in (Ca^2+^)_c_ is an early event in M. smegmatis infection

The role of Ca^2+^ in mycobacterial pathogenesis has been well documented and we were interested to know whether *M*. *smegmatis* altered (Ca^2+^)_c_ levels. Towards this end, HKM were infected with *M*. *smegmatis* and the changes in (Ca^2+^)_c_ levels monitored using Fluo-3/AM. It was observed that infection with *M*. *smegmatis* led to increase in (Ca^2+^)_c_ levels starting at 15 min (p < 0.05) after addition of bacteria with peak level recorded at 1 h; thereafter the levels gradually declined (Supplementary Fig. 2) suggesting the surge in (Ca^2+^)_c_ to be an early event in *M*. *smegmatis* infection. Based on these results we selected 1 h time interval for further assays.

**Figure 4:**
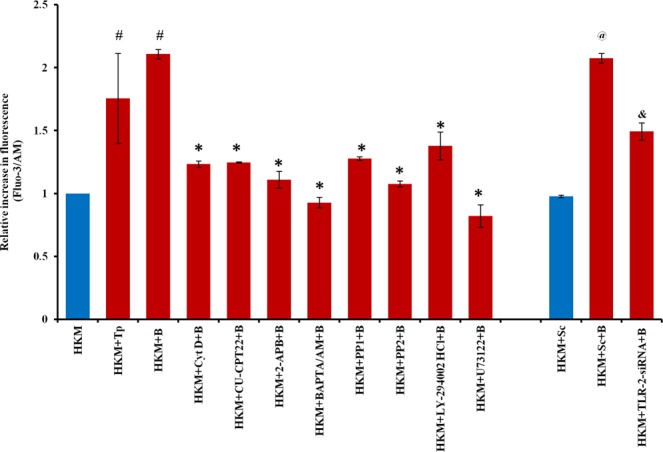
TLR-2 induces (Ca^2+^)_c_ surge. HKM pre-treated with specific inhibitors or transfected with TLR-2-siRNA were infected with *M*. *smegmatis* and increase in (Ca^2+^)_c_ measured using Fluo3/AM. Vertical bars represent mean ± S.E.M (n = 3). ^#^*P* < 0.05 compared to HKM; ^*^P < 0.05 compared to HKM + B; ^@^*P* < 0.05 compared to HKM + Sc; ^**&**^*P* < 0.05 compared to HKM + Sc + B. HKM^,^ uninfected HKM; HKM + Tp, HKM treated with Tp; HKM + B, HKM infected with *M*. *smegmatis*; HKM + Cyt D + B, HKM + CU-CPT22 + B, HKM + 2-APB + B, HKM + BAPTA/AM + B, HKM + PP1 + B, HKM + PP2 + B, HKM + LY-294002HCl + B, HKM + U73122 + B, HKM pre-treated with Cyt D, CU-CPT22, 2-APB, BAPTA/AM, PP1, PP2, LY-294002HCl, U73122 respectively and infected with *M*. *smegmatis*; HKM + Sc, HKM transfected with sc-siRNA; HKM + Sc + B, HKM + TLR-2-siRNA + B, HKM transfected with sc-siRNA, TLR-2-siRNA respectively and infected with *M*. *smegmatis*.

Next, HKM were pre-treated with IP3R inhibitor 2-APB, and alterations in (Ca^2+^)_c_ levels monitored. We observed that pre-treatment with 2-APB significantly reduced *M*. *smegmatis-*induced (Ca^2+^)_c_ surge (Fig. 4), the number of Hoechst 33342 positive cells and attenuated caspase-3 activity (Fig. 2d). It was also observed that Cyt D pre-treatment inhibited *M*. *smegmatis*-induced (Ca^2+^)_c_ surge (Fig. 4) suggesting the importance of phagocytosis in triggering the process of (Ca^2+^)_c_ surge. Tp and BAPTA/AM was used as positive and negative control respectively in the study. Taken together, these results suggested pro-apoptotic involvement of (Ca^2+^)_c_ in *M*. *smegmatis*-induced HKM pathology and the (Ca^2+^)_c_ surge to be primarily from intracellular sources.

We were interested to study the role of TLR-2 in triggering (Ca^2+^)_c_ surge in infected HKM. The cells were pre-treated with or without CU-CPT22 or transfected with TLR-2-siRNA separately and the changes in (Ca^2+^)_c_ levels measured. There was significant reduction in *M*. *smegmatis*-induced alteration in (Ca^2+^)_c_ levels in presence of both CU-CPT22 and TLR-2-siRNA (Fig. 4) clearly indicating the role of TLR-2 in *M*. *smegmatis*-induced (Ca^2+^)_c_ surge from intracellular sources.

We questioned the role of intermediate molecules in TLR-2 mediated (Ca^2+^)_c_ surge. Earlier studies suggested c-Src-PI3K axis plays role in TLR-2 triggered release of (Ca^2+^)_c_ from intracellular sources^17^. HKM were pre-treated with c-Src inhibitors PP1 and PP2 and (Ca^2+^)_c_ surge monitored. We observed that *M*. *smegmatis-*induced (Ca^2+^)_c_ surge was significantly reduced in presence of both PP1 and PP2. To study the role of PI3K in TLR-2 mediated (Ca^2+^)_c_ HKM were pre-treated with PI3K inhibitor LY294002 and changes in (Ca^2+^)_c_ surge monitored. There was significant inhibition in *M*. *smegmatis-*induced (Ca^2+^)_c_ surge in LY294002 pre-treated cells. Among several other molecules PLC has been reported to be involved in TLR-2 activation^17^. In line with this, it was observed that PLC inhibitor U-73122, blocked (Ca^2+^)_c_ surge. Together, our results suggested the intermediary role of c-Src, PI3K and PLC in TLR-2 mediated (Ca^2+^)_c_ surge from intracellular sources in *M*. *smegmatis*-infected cells (Fig. 4).

### TLR-2- induced (Ca^2+^)_c_ triggers ER-stress in HKM

**Figure 5:**
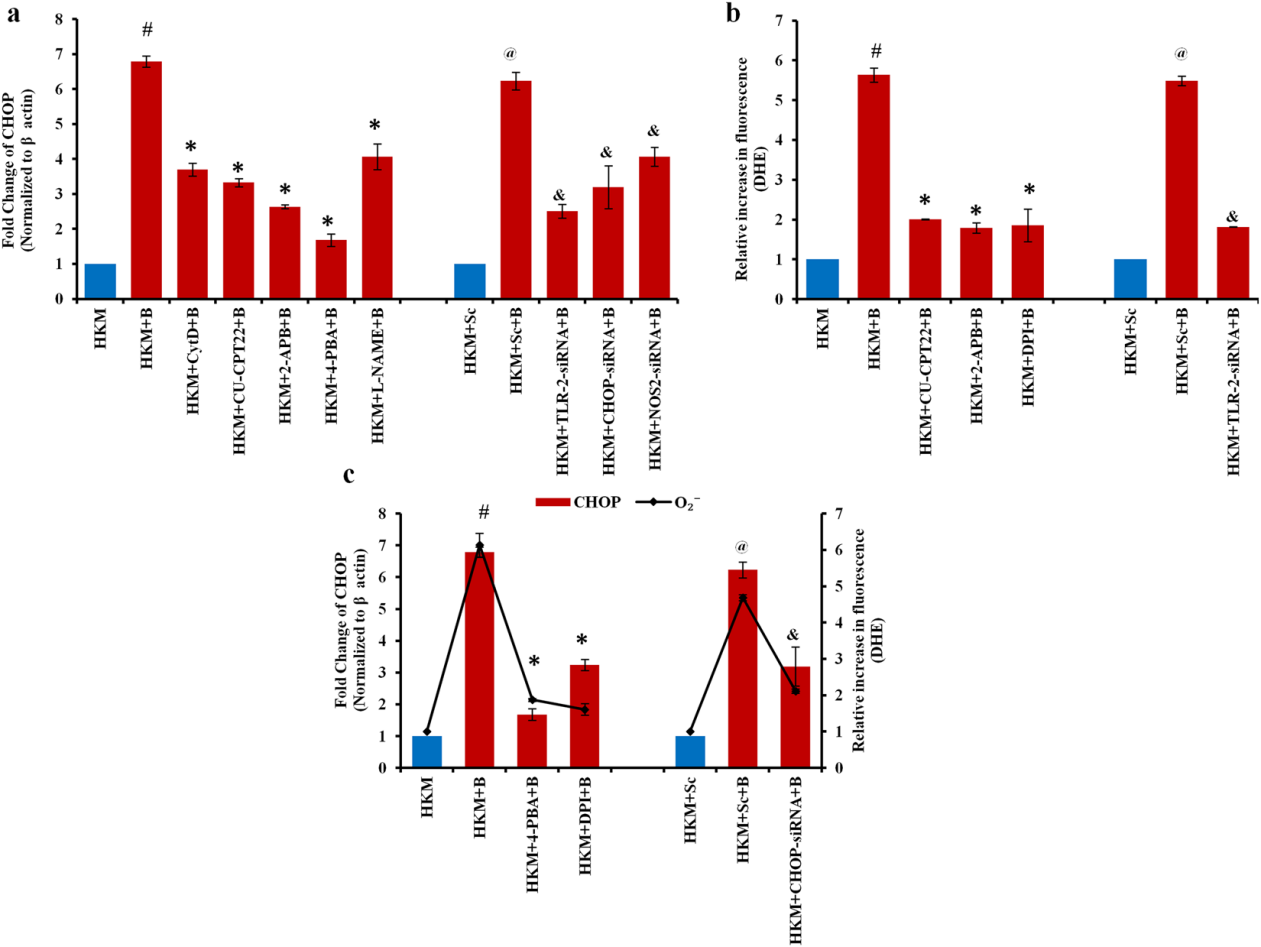
*M*. *smegmatis* induces ER-stress and O_2_^−^ crosstalk in HKM. HKM pre-treated with specific inhibitors or transfected with siRNAs were infected with *M*. *smegmatis* and (**a**) fold change in CHOP-mRNA and (**b**) O_2_^−^ generation measured at 2 h p.i. (**c**) Fold change in CHOP-mRNA and O_2_^−^ generation measured in infected HKM pre-treated with DPI, 4-PBA, and CHOP-siRNA respectively. Vertical bars represent mean ± S.E.M (n = 3). ^**#**^*P* < 0.05 compared to HKM; ^*^P < 0.05 compared to HKM + B; ^**@**^*P* < 0.05 compared to HKM + Sc; ^&^*P* < 0.05 compared to HKM + Sc + B. HKM^,^ uninfected HKM; HKM + B, HKM infected with *M*. *smegmatis*; HKM + Cyt D + B, HKM + CU-CPT22 + B, HKM + 2-APB + B, HKM + 4-PBA + B, HKM + L-NAME + B, HKM pre-treated with Cyt D, CU-CPT22, 2-APB, 4-PBA, L-NAME respectively and infected with *M*. *smegmatis*. HKM + Sc, HKM transfected with sc-siRNA; HKM + Sc + B, HKM + TLR-2-siRNA + B, HKM + CHOP-siRNA + B, HKM + NOS2-siRNA + B HKM transfected with sc-siRNA, TLR-2-siRNA, CHOP-siRNA, NOS2-siRNA respectively and infected with *M*. *smegmatis*.

*M*. *smegmatis*-induced ER-stress has been recently reported in mammalian macrophages^19^. The release of Ca^2+^ from intracellular sources following *M*. *smegmatis* infection prompted us to study ER-stress in the infected HKM. We checked the expression of ER-stress marker CHOP by real-time PCR and observed significant fold increase at 2 h p.i. and thereafter the levels though declined remained significant till 24 h p.i. (Supplementary Fig. 3) clearly demonstrating *M*. *smegmatis* infection induced ER-stress in HKM. We selected 2 h time interval for monitoring CHOP expression in subsequent studies. The HKM pre-treated with Cyt D were infected with *M*. *smegmatis* and CHOP-mRNA expression monitored at 2 h p.i. We observed significant inhibition in CHOP-mRNA expression in Cyt D pre-treated HKM suggesting the importance of phagocytosis in triggering ER-stress (Fig. 5a).

We hypothesised that the release of ER-Ca^2+^ prelude to ER-stress in *M*. *smegmatis* infection. To prove this, HKM were pre-treated with 2-APB, then infected with *M*. *smegmatis* and CHOP-mRNA expression (2 h p.i.) and HKM apoptosis (24 h p.i.) studied. We observed that CHOP-mRNA expression (Fig. 5a) and HKM apoptosis (Fig. 2d) were alleviated in presence of 2-APB suggesting positive correlation between depletion of ER-Ca^2+^ and induction of pro-apoptotic ER-stress in *M*. *smegmatis* infection. General ER-stress inhibitor 4-PBA and CHOP-siRNA significantly down-regulated CHOP expression (Fig. 5a) with concomitant decline in caspase-3 activity and HKM apoptosis (Fig. 2d). Collectively, these results demonstrated that *M*. *smegmatis*-induced ER-stress leads to HKM apoptosis.

We concluded by studying the role of TLR-2 in ER-stress. HKM were pre-treated with CU-CPT22 or transfected with TLR-2-siRNA and the changes in CHOP expression studied. There was significant reduction in CHOP expression implicating the role of TLR-2 signalling in *M*. *smegmatis*-induced ER-stress (Fig. 5a).

### TLR-2 signalling induces O_2_^−^ stress in *M*. *smegmatis*-infected HKM

ROS plays important role in mycobacterial pathogenesis^15,21^. To study its role in *M*. *smegmatis* infection, we used specific dye, DHE and found significant release of ROS in infected HKM with peak levels recorded at 2 h p.i.; thereafter though the levels declined but remained higher than uninfected HKM (Supplementary Fig. 4). We selected 2 h time interval for subsequent studies on ROS production. The HKM were pre-treated with NADPH oxidase inhibitor DPI^33^ then infected with *M*. *smegmatis* and ROS levels measured using DHE. We observed significantly reduced ROS levels in presence of DPI (Fig. 5b) suggesting the species of ROS generated during *M*. *smegmatis* infection to be primarily O_2_^−^. Pre-treatment of HKM with 2-APB significantly reduced O_2_^−^ levels (Fig. 5b), caspase-3 activity and HKM apoptosis (Fig. 2d) demonstrating the role of ER-Ca^2+^ in O_2_^−^ stress along with pro-apoptotic implication.

In parallel study, when HKM were pre-treated with CU-CPT22 or transfected with TLR-2-siRNA, there was significant reduction in *M*. *smegmatis*-induced O_2_^−^ levels (Fig. 5b). Together, our results for the first time implicated the role of TLR-2 in governing two important molecular events, ER-stress and O_2_^−^ generation during *M*. *smegmatis*-infection.

As induction of ER-stress and O_2_^−^ production was occurring simultaneously, we were interested to study the crosstalk between these two events. To look into this, HKM were transfected with CHOP-siRNA or pre-treated with 4-PBA or DPI and *M*. *smegmatis*-induced O_2_^−^ generation and ER-stress monitored respectively. We observed significant reduction in O_2_^−^ levels in 4-PBA pre-treated and CHOP-siRNA transfected-infected HKM. Concurrently, there was significant down-regulation in CHOP-mRNA levels in HKM pre-treated with DPI (Fig. 5c) which suggested that the crosstalk between ER-stress and O_2_^−^ production to be important in *M*. *smegmatis*-induced pathogenesis.

### ER-stress O_2_^−^ crosstalk amplifies TNF-α production

**Figure 6:**
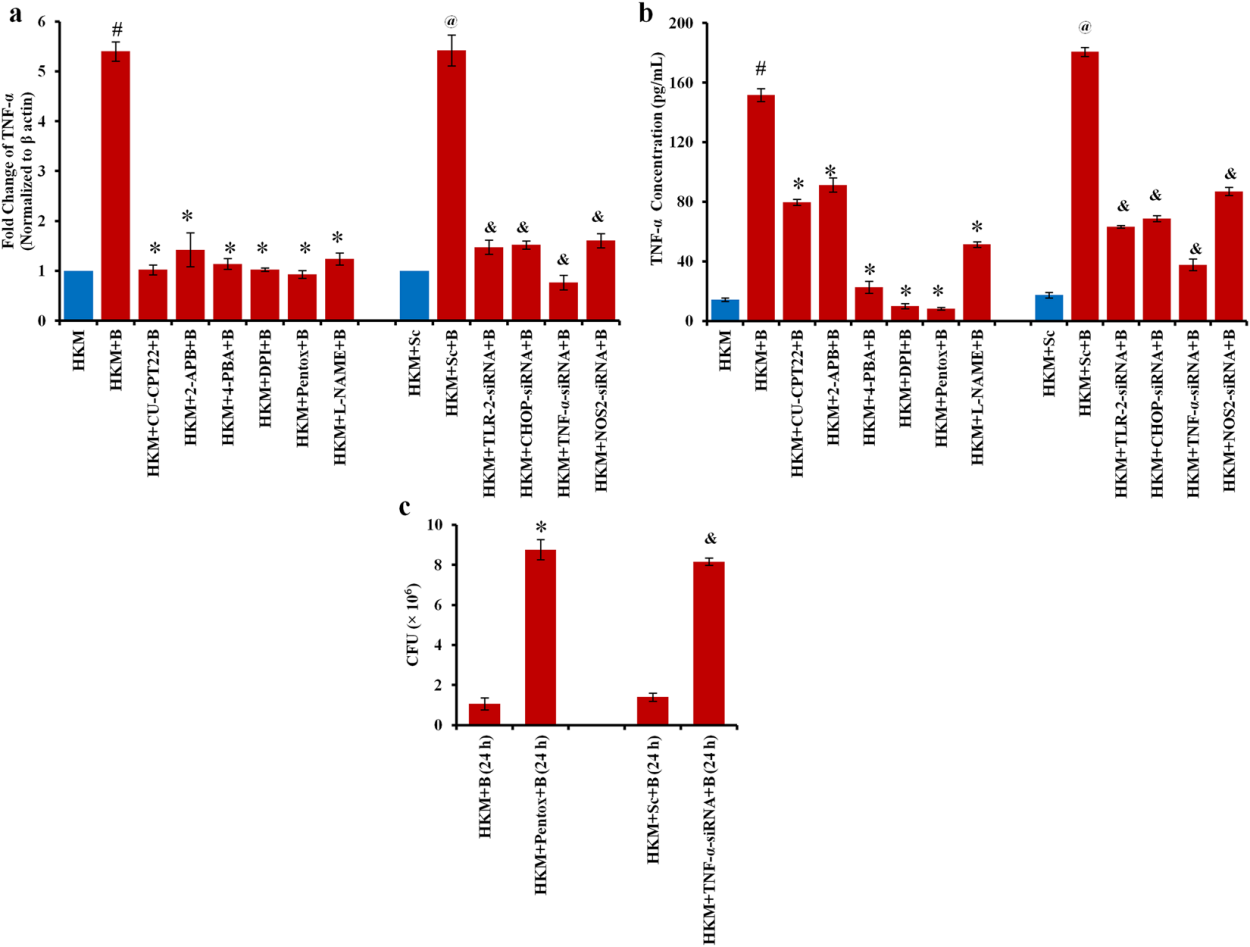
ER-stress-O_2_^−^ cross-talk amplifies TNF-α production. HKM pre-treated with indicated inhibitors or transfected with specific siRNAs were infected with *M*. *smegmatis* and (**a**) fold change in TNF-α-mRNA (6 h p.i) (**b**) production of TNF-α protein (24 h p.i.) quantified (**c**) viability of intracellular bacteria enumerated at 24 h p.i. Vertical bars represent mean ± S.E.M (n = 3). ^#^*P* < 0.05 compared to HKM; ^*^P < 0.05 compared to HKM + B and HKM + B (24 h); ^@^*P* < 0.05 compared to HKM + Sc; ^&^*P* < 0.05 compared to HKM + Sc + B and HKM + Sc + B (24 h). HKM, uninfected HKM; HKM + B, HKM infected with *M*. *smegmatis*; HKM + CU-CPT22 + B, HKM + 2-APB + B, HKM + 4-PBA + B, HKM + DPI + B,, HKM + Pentox + B, HKM + L-NAME + B, HKM pre-treated with CU-CPT22, 2-APB, 4-PBA, DPI, Pentox, L-NAME respectively and infected with *M*. *smegmatis*; HKM + Sc, HKM transfected with sc-siRNA; HKM + Sc + B, HKM + TLR-2-siRNA + B, HKM + CHOP-siRNA + B, HKM + TNF-α-siRNA + B, HKM + NOS2-siRNA + B, HKM transfected with sc-siRNA, TLR-2-siRNA, CHOP-siRNA, TNF-α-siRNA, NOS2-siRNA respectively and infected with *M*. *smegmatis*.

The role of TNF-α in containing mycobacterial infections is well reported^34^. The gradual clearance of intracellular bacteria (Fig. 3b) encouraged us to check whether *M*. *smegmatis* infection induced TNF-α production. The changes in TNF-α-mRNA expression were studied and we observed significant up-regulation with peak level recorded at 6 h p.i. (Supplementary Fig. 5). We extended the study by monitoring changes in cytokine levels using specific ELISA kit and observed maximum production of TNF-α at 24 h p.i. (Supplementary Fig. 6). TNF-α (mRNA and protein) production was attenuated in presence of CU-CPT22, TLR-2-siRNA, 2-APB and pentox (Fig. 6a,b). Together these results helped in establishing the role of TLR-2 induced release of Ca^2+^ from intracellular sources on TNF-α production.

We further observed that TNF-α levels were significantly reduced on pre-treatment with 4-PBA and DPI clearly implicating cross-talk between ER-stress and O_2_^−^ impacts the release of TNF-α in *M*. *smegmatis*-infected HKM (Fig. 6a,b).

We concluded the study by inhibiting TNF-α and monitoring intracellular bacterial load and HKM apoptosis. It was noted pre-treatment with pentox or TNF-α-siRNA led to increase in intracellular bacteria (Fig. 6c) with concomitant decline in HKM apoptosis (Fig. 2d) suggesting the role of TNF-α in regulating intracellular load of *M*. *smegmatis* and HKM apoptosis.

### Activation of O_2_^−^NO axis helps in clearance of intracellular *M*. *smegmatis*

**Figure 7:**
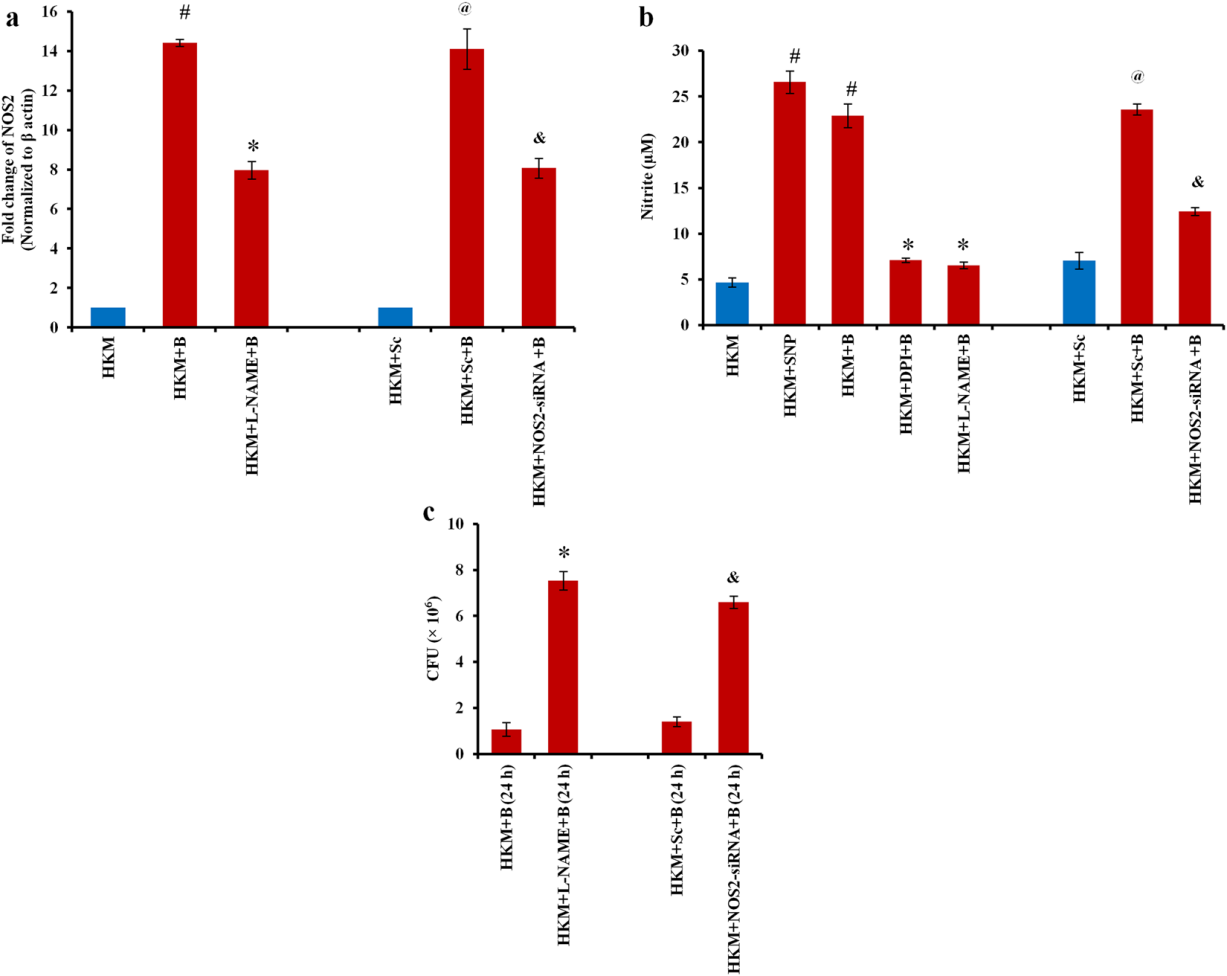
*M*. *smegmatis-*induced NO is bactericidal. HKM were pre-treated with or without DPI, L-NAME separately, transfected with sc-siRNA or NOS2-siRNA prior to infection and (**a**) fold change in NOS2-mRNA expression measured and (**b**) NO levels quantified 24 h p.i. (**c**) Intracellular bacteria enumerated at 24 h p.i. Vertical bars represent mean ± S.E.M (n = 3). ^**#**^*P* < 0.05 compared to HKM; ^*^P < 0.05 compared to HKM + B and HKM + B (24 h); ^**@**^*P* < 0.05 compared to HKM + Sc; ^**&**^*P* < 0.05 compared to HKM + Sc + B and HKM + Sc + B (24 h). HKM, uninfected HKM; HKM + SNP, HKM treated with SNP; HKM + B, HKM infected with *M*. *smegmatis*; HKM + DPI + B, HKM + L-NAME + B, HKM pre-treated with DPI, L-NAME respectively and infected with *M*. *smegmatis*; HKM + Sc, HKM transfected with sc-siRNA; HKM + Sc + B, HKM + NOS2-siRNA + B, HKM transfected with sc-siRNA, NOS2-siRNA respectively and infected with *M*. *smegmatis*.

Macrophages respond to mycobacterial infection by NOS2-mediated NO production^15,35^. We checked NOS2-mRNA expression and NO production in HKM consequent to *M*. *smegmatis* infection. There was significant upregulation of NOS2-mRNA from 2 h p.i. with peak level recorded at 24 h p.i. (Supplementary Fig. 7). NO production was found to be highest at 24 h p.i. (Supplementary Fig. 8). Pre-treatment of HKM with NOS2-siRNA and NO inhibitor, L-NAME significantly reduced NOS2-mRNA expression (Fig. 7a) and NO production (Fig. 7b). SNP was used as the positive control and induced significant NO production in uninfected HKM. The next step was studying the role of NO and we observed that inhibiting NOS2-NO axis using L-NAME or NOS2-siRNA led to significant increase in intracellular *M*. *smegmatis* (Fig. 7c) coupled with decrease in caspase-3 activity and HKM apoptosis (Fig. 2d) clearly suggesting bactericidal and pro-apoptotic role of NO in *M*. *smegmatis*-infected HKM. The presence of L-NAME in the culture medium did not influence bacterial growth *per se* (data not shown).

Our next step was identifying upstream signals influencing NO release. O_2_^−^ has been implicated in the production of RNI^26,36^. To study this, HKM pre-treated with DPI were infected with *M*. *smegmatis* and the changes in NO production monitored. There was significant reduction in *M*. *smegmatis*-induced NO production in DPI pre-treated-infected HKM compared to untreated-*M*. *smegmatis* infected (Fig. 7b). The reverse was not true as pre-treatment with L-NAME had no significant effect on *M*. *smegmatis*-induced O_2_^−^ levels (Supplementary Fig. 9). From these observations, we concluded that O_2_^−^ influenced the production of NO in *M*. *smegmatis-*infected HKM.

NO impacts ER-stress^37^ and TNF-α production^38^. We extended our study monitoring the cross-talk of NO with ER-stress and TNF-α production respectively. It was observed that inhibiting NO production with L-NAME or specific siRNA attenuated CHOP expression (Fig. 5a) and TNF-α production (Fig. 6a,b). Conversely, 4-PBA and pentox had no significant effect on the release of NO in infected HKM (Supplementary Fig. 10). Together, our results suggested the role of nitrosative stress on ER-stress and production of TNF-α in *M*. *smegmatis*-infected HKM.

## Discussion

Ability to induce host cell pathology depends on dose of the infecting agent^39^. Interestingly, we observed *M*. *smegmatis-*induced HKM death to be MOI dependent suggesting bacterial load as a major contributor to *M*. *smegmatis*-induced host cell cytotoxicity that has not been reported earlier. The implication of mycobacterial infections on host cell death is inconclusive. There are reports suggesting mycobacteria induce host cell apoptosis, whereas in others it inhibits the process and grow successfully in macrophages^40^. Recently, few observations have also suggested that mycobacteria-induced cell death shares features of both apoptotic and necrotic cell death^41^.

We observed presence of oligonucleosomal DNA ladder, intensely stained Hoechst positive and AV positive cells confirming that *M*. *smegmatis* prompts apoptosis in HKM. Apoptosis due to mycobacterial infection can be caspase-dependent^42^ or –independent^43^. Caspase-3 dependent apoptosis was observed in human and murine macrophages infected with *M*. *smegmatis*^19,22^ but the same was not reported in fish. We observed significant caspase-3 activity and inhibiting the protease led to decline in HKM death confirming *M*. *smegmatis*-induced HKM apoptosis to be caspase-mediated. Our results strongly suggest the potential of *M*. *smegmatis* to induce caspase mediated apoptosis is conserved among diverse hosts.

Pathogens encounter different microenvironments during the course of infection to which they rapidly adapt to induce pathogenesis. One way by which pathogens achieve this is by regulating the expression of virulence factors. Earlier reports suggest that the pathogenic potential differs during different phases of bacterial growth^44^. ESAT-6 and CFP-10 are well known mycobacterial virulence factors which are also conserved and functional in *M*. *smegmatis*^45^. We assumed a positive co-relation between virulence gene expression and HKM apoptosis and observed the late log phase isolates to be most cytotoxic with maximum expression of both virulence factors. Our findings are in concordance with earlier studies^46,47^ suggesting virulence related proteins to be growth phase dependent in mycobacteria.

The significance of bacterial load in HKM apoptosis prompted us to study the mechanism of phagocytosis and its subsequent effects. We observed that HKM efficiently phagocytosed *M*. *smegmatis* triggering gradual death of the intracellular bacterium and in the process itself underwent apoptosis. This is in disparity to an earlier study proposing that mammalian macrophages undergo cycles of high and low activity with respect to killing of *M*. *smegmatis* but do not die^48^. We believe these variances are due to differences in the nature of cells (mammalian *vs* fish), culture conditions and MOI used in the two studies.

The role of TLR-2 in mycobacterial recognition and phagocytosis is well established^12^ but the same has not been implied in fish. We observed significant upregulation of TLR-2 and its adaptor MyD88 and inhibiting TLR-2 signalling compromised uptake of *M*. *smegmatis*. In an earlier report upregulation of TLR-2 expression in *M*. *marinum*-infected zebrafish has been documented^49^. With the consensus of earlier report and our observation, we conclude that the role of TLR-2 in innate recognition of mycobacteria is conserved across species. However, unlike earlier studies reporting the ligation of TLR-2 with extracellular mycobacteria or its components sufficient for triggering host cell apoptosis, we suggest that the engagement of membrane bound TLR-2 and internalisation of metabolically active *M*. *smegmatis* is essential for inducing HKM apoptosis. Although, based on these observations, it is not possible to ascertain whether TLR-2 *per se* has direct role in phagocytosis of *M*. *smegmatis* or acts by stimulating phagocytic receptors. Nonetheless, linking TLR-2 in innate recognition and phagocytosis of *M*. *smegmatis* by HKM has been reported for the first time in the present manuscript. Our results are of great importance as TLRs are crucial in the recognition of mycobacteria and could have an impact on host responses.

The participation of Ca^2+^ as a second messenger in TLR-2 dependent signalling is essential component of many cellular signalling cascades^50^. The involvement of TLR-2 in triggering (Ca^2+^)_c_ surge has not been reported in *M*. *smegmatis* infection. Moreover, the role of (Ca^2+^)_c_-surge in mycobacterial-pathogenesis lack clarity with reports suggesting positive as well negative roles on mycobacterial survival and immunity^40^. *M*. *smegmatis* infection elevated (Ca^2+^)_c_ level and inhibiting TLR-2 signalling and IP3R channels abrogated the surge and attenuated HKM apoptosis. Together, these results demonstrate TLR-2 as conduit linking bacterial stimuli with release of Ca^2+^ from intracellular sources augmenting apoptosis of infected cells.

It has been reported that Src family kinases together with PI3K and PLC influence TLR-2 mediated release of (Ca^2+^)_c_ from intracellular sources^17^. Using combination of inhibitors we found intermediary role of c-Src-PI3K-PLC axis in TLR-2 mediated release of (Ca^2+^)_c_ through IP3R in *M*. *smegmatis*-infected HKM. To best of our knowledge, this is the first report linking TLR-2 with (Ca^2+^)_c_ surge in *M*. *smegmatis* infection activating downstream apoptotic signals. Based on our results, we put forward that TLR-2-mediated alteration in (Ca^2+^)_c_ level acts as innate immune mechanism that triggers downstream signals to help host overcome perceived threat of infection.

Altered (Ca^2+^)_c_ levels trigger several cellular events including ER-stress^18^ and ROS generation^51^. Though, pro-apoptotic involvement of both ER-stress and ROS has been reported in *M*. *smegmatis*^19^, the upstream molecular events still remain to be identified. We sought to link TLR-2 signalling with ER-stress and ROS generation in *M*. *smegmatis*-infected cells. CHOP expression is signature of ER-stress and several reports evidenced its role in mycobacteria-induced apoptosis^52^. We observed significant increase in CHOP-mRNA expression and inhibiting TLR-2 signalling or blocking IP3R channels attenuated CHOP-mRNA expression and HKM apoptosis. This clearly established the role of TLR-2 induced (Ca^2+^)_c_ surge from intracellular sources in triggering ER-stress and apoptosis of *M*. *smegmatis-*infected HKM. As a connect with earlier study^19^, we also observed that inhibiting phagocytosis down-regulated CHOP expression reconfirming intracellular load is crucial for *M*. *smegmatis* pathogenesis.

In addition, we observed significant O_2_^−^ generation which was otherwise inhibited in absence of TLR-2 and IP3R mediated signals implicating the role of TLR-2 mediated (Ca^2+^)_c_ surge in oxidative stress and apoptosis of *M*. *smegmatis-*infected HKM. Our results suggested that both ER-stress and ROS were contributing towards *M*. *smegmatis-*induced HKM pathology. Interestingly, when we compared the kinetics of the two distinct apoptotic signals we noted considerable overlap, possibly due to cross talk. It is evident from our results that inhibiting ER-stress led to corresponding decline in O_2_^−^ levels and interference with O_2_^−^ generation ameliorated ER-stress implicating dual role of TLR-2 in regulating ER-stress and O_2_^−^ generation. Since there is no published report till date related to this key aspect, we propose that the ER-stress-O_2_^−^ cross-talk aggravates *M*. *smegmatis*-induced HKM pathology.

Further, to identify the downstream target of this cross-talk, we observed time dependent increase in TNF-α levels and inhibiting the cytokine resulted in increased intracellular bacterial load, reduction in caspase-3 activity and HKM apoptosis. The role of pro-inflammatory cytokine TNF-α in containing virulent^34^ and avirulent mycobacteria^19^ is well established. There are reports suggesting the involvement of both ER-stress^53^ and ROS^54^ on TNF-α production. Akin to this, we observed that ameliorating either ER-stress or O_2_^−^ generation in *M*. *smegmatis*-infected HKM markedly affected TNF-α production highlighting non-redundant and synergistic roles of the two molecular events in production of the pro-inflammatory cytokine. We propose that the cross-talk between ER-stress-O_2_^−^ amplifies production of TNF-α, essential for removal of *M*. *smegmatis* and triggering host cell apoptosis.

The role of RNI in mycobacterial infections is not very clear as it has been reported to have both pro- and anti-apoptotic effects^55^. We observed distinct bactericidal and pro-apoptotic role of NO in the present study. It was also noted that inhibiting O_2_^−^ generation affects NO level but on the contrary, failed to observe any appreciable effect of RNI inhibition on O_2_^−^ generation. This suggests that besides pro-inflammatory cytokine production O_2_^−^ also helps in conversion of NO to ONOO^−^, a potent bactericidal agent in infected HKM. Our results are in conjunction with earlier studies suggesting combined effect of O_2_^−^ and NO helps in determining survival of *M*. *smegmatis* in human macrophages^56^.

It has been reported that NO plays role in ER-stress^37^ and pro-inflammatory cytokine production^38^. We observed that inhibiting NO production affected CHOP expression and TNF-α production but the reverse was not true. We posit that late production of NO helps in prolonging ER-stress and TNF-α production, thereby together triggering clearance of *M*. *smegmatis* and host cell apoptosis.

**Figure 8:**
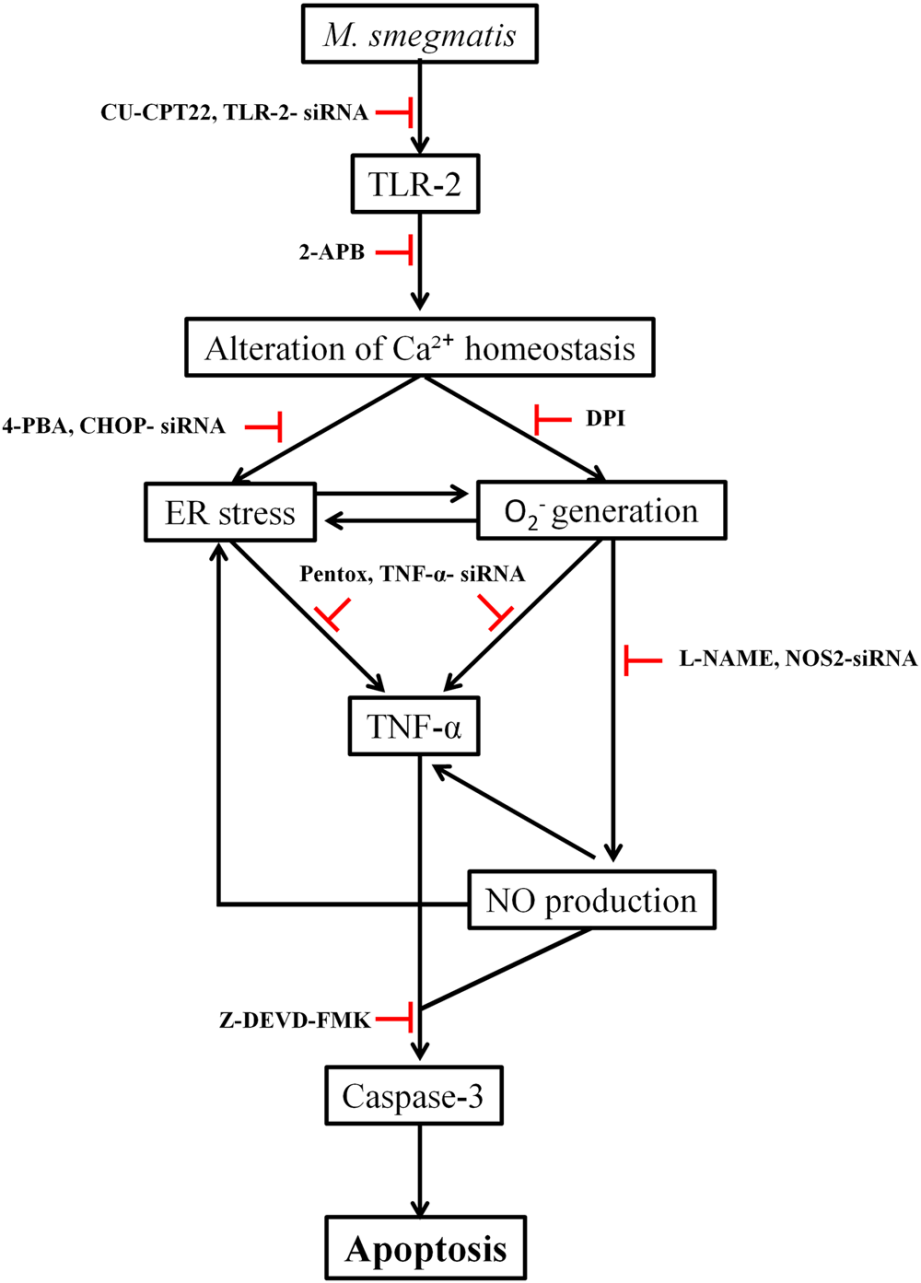
Overview of the work. TLR-2 signalling induces (Ca^2+^)_c_ surge triggering ER-stress and O_2_^−^ generation. The cross-talk between ER-stress and O_2_^−^ amplifies TNF-α levels. O_2_^−^ contributes to nitrosative stress that prolongs ER-stress and TNF-α production. The cascade leads to caspase-mediated apoptosis of infected HKM and clearance of *M*. *smegmatis*.

To conclude, our results zealously suggest that *M*. *smegmatis* induces TLR-2 mediated (Ca^2+^)_c_ surge causing ER-stress and generation of O_2_^−^ in HKM. The cross-talk between these two events amplifies production of pro-inflammatory TNF-α. Additionally, O_2_^−^ contributes to nitrosative stress which in turn helps in protracting ER-stress and TNF-α production. The cascade of events lead to caspase mediated HKM apoptosis and bacterial clearance (Fig. 8). Thus, HKM not only provides comprehensive view of *M*. *smegmatis-*macrophage interactions it also mimics several aspects of mammalian macrophage responses to mycobacteria, suggesting it a convenient alternate model to study host-mycobacterial interactions.

## Materials and Methods

### Ethics statement

Animal experiments described in this study were approved by the Animal Ethics Committee, University of Delhi (DU/ZOOL/IAEC-R/2013/34) and carried out in accordance with the protocols approved by The Prevention of Cruelty to Animals Act, Govt. of India.

### Animal care and maintenance

*C*. *gariepinus* (80–100 g) were purchased from local fish firm and kept in 50 L glass tanks. Fish were fed boiled chicken liver *ad libitum* and acclimatized to laboratory conditions for 15 d before use for experimental purposes. The water quality and fish health in tanks was regularly monitored^57^.

### Bacterial preparation

*M*. *smegmatis* mc^2^ 155 was a kind gift from William R. Jacobs Jr, Albert Einstein College of Medicine, New York. The bacteria were grown in Middlebrook 7H9 broth medium (HiMedia) at 30 °C supplemented with 10% albumin, dextrose, catalase (ADC), 0.05% Tween-80, 0.50% glycerol, 100 µg/mL ampicillin with aeration in shaking incubator at 120 rpm. Antibiogram suggested the isolate to be sensitive to amikacin and resistant to ampicillin and cefalexin respectively. For some experiments, *M*. *smegmatis* were heat killed (30 min at 70 °C), treated with amikacin (50 µg/mL for 1 h) or formalin fixed (1% formaldehyde at 4 °C overnight) and washed thoroughly in PBS. The viability of bacteria thus prepared was checked by dilution plating on Middlebrook 7H11 agar plates.

### HKM isolation and infection

The method for isolation of HKM has been described earlier^32^. Aseptically removed headkidneys were made into single cell suspension using 100 µm wire mesh (Sigma) and layered on 34/51% percoll density gradient. The suspension was centrifuged at 400 × g for 20 min at 4 °C and the fraction rich in phagocytes that appear above the 34/51 interface was collected, washed and enriched for macrophages. Wright Giemsa staining was done to check the purity of the HKM and viability determined by 0.4% trypan blue dye exclusion method^58^.

HKM were washed in antibiotic-free RPMI-1640 supplemented with 10% FBS, distributed in 24 well tissue culture plates, then infected with *M*. *smegmatis* with indicated multiplicity of infection (MOI) and incubated for 4 h at 30 °C under 5% CO_2_. After 4 h, the infected cells were treated with amikacin (50 µg/mL) for 1 h to kill extra-cellular bacteria with no effect on HKM viability. This time point was taken as 0 h post infection (p.i.). Finally, the infected HKM were washed, re-suspended in RPMI-1640 supplemented with FBS (10%), pen-strep (1%) (complete-RPMI) and maintained at 30 °C under 5% CO_2_ for further studies.

### Chemicals and kits

Caspase-3 inhibitor (Z-DEVD-FMK, 10 µM) was purchased from Biovision. TLR-2 inhibitor (CU-CPT22, 1 µM), actin polymerisation inhibitor cytochalasin D (Cyt D, 5 µM), intracellular Ca^2+^chelator 1, 2-Bis (2-aminophenoxy) ethane-N,N,N0,N0-tetraacetic acid tetrakis/acetoxymethyl ester (BAPTA/AM, 5 mM), IP3 receptor antagonist 2-Aminoethyl diphenylborinate (2-APB, 100 µM), selective inhibitors of Src family protein tyrosine kinases 4-Amino-5-(methylphenyl)-7-(t-butyl)pyrazolo-(3,4-d) pyrimidine (PP1, 25 µM), 4-Amino-5-(4-chlorophenyl)-7-(t-butyl)pyrazolo[3,4-d]pyrimidine (PP2, 25 µM), PI3Kinase inhibitor (LY-294002 hydrochloride, 14.5 µM), NADPH oxidase inhibitor diphenyleneidonium chloride (DPI, 10 µM), general ER-stress inhibitor 4-phenyl butyric acid (4-PBA, 10 µM), TNF-α biosynthesis inhibitor pentoxifylline (Pentox, 1 mM), Nω-Nitro-L-arginine methyl ester hydrochloride (L-NAME, 1 mM), apoptosis inducer staurosporine (STS, 1 µM), TLR-2 agonist (Pam3CSK4, 50 µg/mL) and inducer of Ca^2+^ release from ER thapsigargin (Tp, 1 µM) were purchased from Sigma. Phospholipase C inhibitor (U73122, 10 µM) was purchased from Calbiochem. Nitric oxide donor sodium nitroprusside (SNP, 2 mM) was purchased from Enzo Life Sc. Treatment of HKM with specific inhibitors or positive controls were done for 1 h prior to infection. The doses of the inhibitors and positive controls were decided based on the specificity and cytotoxic effects on HKM. The indicated doses did not induce cytotoxic effects on HKM and were maintained throughout the course of experiments.

FITC Annexin V apoptosis detection kit, first strand cDNA synthesis kit, fish TNF-α kit, Griess reagent kit, caspase-3 colorimetric assay kit were purchased from BD Pharmingen, MBI Fermentas, MyBiosource, Invitrogen and Biovision respectively.

### Phagocytosis and enumeration of intracellular bacteria

HKM (2 × 10^6^) pre-treated with indicated concentrations of respective inhibitors and transfected with non-targeting scrambled siRNA (sc-siRNA) or targeted specific siRNAs were infected with *M*. *smegmatis*. At indicated time p.i., HKM were lysed with 0.1% Triton X-100, and 20 µL MTT (3,-(4, 5- Dimethylthiazol-2-yl) – 2, 5 diphenyltetrazolium bromide) (Merck) from stock of 5 mg/mL was added and incubated for 5 h. Following incubation, the formazan crystals thus formed were dissolved in dimethyl sulfoxide (DMSO), absorbance read in a microplate reader (Epoch2, BioTek) at A_595._ The number of intracellular bacteria was enumerated by interpolating the absorbance of samples from the standard curve^59^.

### Real-time PCR

HKM (2 × 10^7^) pre-treated with or without inhibitors or transfected with sc-siRNA or specific siRNAs were harvested at indicated time points p.i., total RNA isolated in TRIZOL (Sigma) and dissolved in diethyl pyrocarbonate (DEPC) water^15^. 1 µg RNA was used as template for preparing cDNA using first strand cDNA synthesis kit^32^. The same protocol was followed to isolate total RNA and prepare cDNA from *M*. *smegmatis* (5 × 10^7^) harvested at different phases of growth.

Real-time primers were designed (Supplementary Table 1) and fold changes in ESAT-6, CFP-10, TLR-2, MyD88, CHOP, TNF-α and NOS2 expression was monitored (ABI ViiA, Applied Biosystems) using SYBR green PCR master mix (Applied Biosystems) as described earlier^15^. The expression of each gene were analysed by comparative ΔΔC_T_ method wherein β-actin was taken as endogenous control for HKM and 16 S rDNA in case of *M*. *smegmatis*.

### siRNA transfection

siRNA transfection was done using HiPerfect Transfection Reagent (Qiagen)^15^. Briefly, 50 nM siRNA and 5 μL HiPerfect was added to 90 μL Opti-MEM (Invitrogen), incubated for 20 min at 30 °C for complex formation which was added to HKM maintained in Opti-MEM and the volume made up to1 mL (final concentration of siRNA 5 nM). The HKM-siRNA complex was incubated at 30 °C under 5% CO_2_ for 16 h and subsequently infected with *M*. *smegmatis*. The list of siRNAs used is given in Supplementary Table 2.

### Measurement of (Ca^2+^)_c_ -surge

HKM (1 × 10^6^) pre-treated with or without inhibitors or transfected with sc-siRNA or specific siRNAs were incubated with 2 µM Fluo-3/AM (Invitrogen) for 1 h in the dark. HKM were washed to remove excess dye, infected with M. *smegmatis* and the changes in fluorescence intensity measured at indicated time points on a microplate fluorimeter (Spectramax M2, Molecular Devices) at excitation A_485_ and emission A_520_^57^. Tp was used as positive control in the assay.

### Measurement of O_2_^−^

The production of O_2_^−^ was measured using DHE (Dihydroethidium, Invitrogen). HKM (1 × 10^6^) pre-treated with or without inhibitors or transfected with sc-siRNA or specific siRNAs were infected with *M*. *smegmatis*, washed at indicated time points p.i. and incubated with 5 μM DHE for 15 min. The intensity of fluorescence was measured in a microplate fluorimeter (Spectramax M2, Molecular Devices) at excitation A_520_ and emission A_580_^15^.

### TNF-α quantification

HKM (1 × 10^6^) pre-treated with or without inhibitors or transfected with sc-siRNA or specific siRNAs were infected with *M*. *smegmatis*. The cell-free culture supernatant was collected 24 h p.i. and TNF-α levels measured with fish specific TNF-α ELISA kit using reagents supplied with the kit as described earlier^57^.

### Measurement of nitric oxide (NO)

HKM (1 × 10^6^) pre-treated with or without indicated inhibitors or transfected with sc-siRNA or specific siRNAs were infected with *M*. *smegmatis*. The cell free supernatants were collected at indicated time points p.i., 50 μL each of supernatant and Griess reagent were added to 96-well plate and incubated for 10 min in dark at room temperature. The absorbance was read in a microplate reader (Epoch2, BioTek) at A_540_ and the amount of nitrite generated was determined from NaNO_2_ standard curve^15^. SNP was used as positive control in the assay.

### Cytotoxicity and Apoptosis study

#### Trypan blue dye exclusion

Uninfected and infected HKM (1 × 10^6^) were collected at indicated time p.i., washed, resuspended in PBS and cytotoxicity determined by 0.4% trypan blue dye exclusion method. Briefly, equal volume of HKM suspension and trypan blue were mixed gently, loaded to a hemocytometer and observed under light microscope (Meiji). Three fields with minimum100 cells were observed to determine percentage HKM cytotoxicity.

#### DNA fragmentation

Uninfected and infected HKM (1 × 10^7^) were collected 24 h p.i., lysed in buffer containing 0.2% Triton X 100, 10 mM Tris (pH 7.2), 1 mM EDTA (pH 8.0), proteinase K (5 mg/mL) and the DNA extracted. The electrophoresis of DNA was done on 1.2% agarose gel alongside DNA marker (Roche) and visualised under UV transilluminator^58^.

#### Hoechst 33342 staining

HKM (1 × 10^6^) pre treated with or without indicated inhibitors or transfected with sc-siRNA or specific siRNAs were infected with *M*. *smegmatis*. HKM were collected 24 h p.i., washed and fixed in 3.7% paraformaldehyde at room temperature. The fixed cells were then stained with Hoechst 33342 (2 μg/mL, Sigma) and observed under fluorescence microscope (Nikon Eclipse 400) within 30 min of staining. Hoechst 33342 staining helps in identifying intensely condensed chromatin in apoptotic cells^60^. For enumeration of Hoechst 33342 positive and negative HKM, at least 100 cells were counted in each field and three such fields were observed^15^.

#### Annexin V-FITC and PI staining

Uninfected and infected HKM (1 × 10^6^) were stained with AV-FITC-PI as described earlier and following manufacturer’s instructions (BD Pharmingen)^15^ The cells were observed under fluorescence microscope (Nikon Eclipse 400) within 30 min of adding the dye. Three fields with 100 cells each were studied to determine the percentage of apoptotic HKM^15^.

#### Caspase-3 assay

Caspase-3 activity was assayed using assay kit following the manufacturer’s instruction using chemicals and reagents supplied with the kit. Briefly, HKM (1 × 10^7^) pre-incubated with or without inhibitors were infected with *M*. *smegmatis* collected 24 h p.i., lysed and the supernatant obtained (50 μl) incubated with caspase-3 specific substrates in reaction buffer for 5 h at 30 °C. The pNA light emission was recorded using a microtiter plate reader at A_405_ nm (Epoch2, BioTek) and relative fold changes in caspase-3 plotted^15^.

### Statistical analysis

Statistical data analysis was done using pair wise comparison with the help of t-test: two samples using unequal variances in order to determine the statistical significance between groups considering P < 0.05 as statistically significant. Mean ± SEM is represented by vertical bars.

## Supplementary information

Supplementary Info
