# Supplementary material for: TLR-2 mediated cytosolic-Ca2+ surge activates ER-stress-superoxide-NO signalosome augmenting TNF-α production leading to apoptosis of Mycobacterium smegmatis-infected fish macrophages
Source: Sci Rep. 2019 Aug 23;9:12330. doi: 10.1038/s41598-019-48847-1 (PMC6707155; doi:10.1038/s41598-019-48847-1)
Supplement: Supplementary file 1 — Supplementary Info [file 41598_2019_48847_MOESM1_ESM.pdf]

Supplementary Information

TLR-2 mediated cytosolic-Ca^{2+} surge activates ER-stress-superoxide-NO signalosome augmenting TNF-α production leading to apoptosis of *Mycobacterium smegmatis* infected fish macrophages.

Md. Arafat Hussain[1]*, Debika Datta[1], Rashmi Singh[1], Manmohan Kumar[1], Jai Kumar[1] and Shibnath Mazumder[1]*

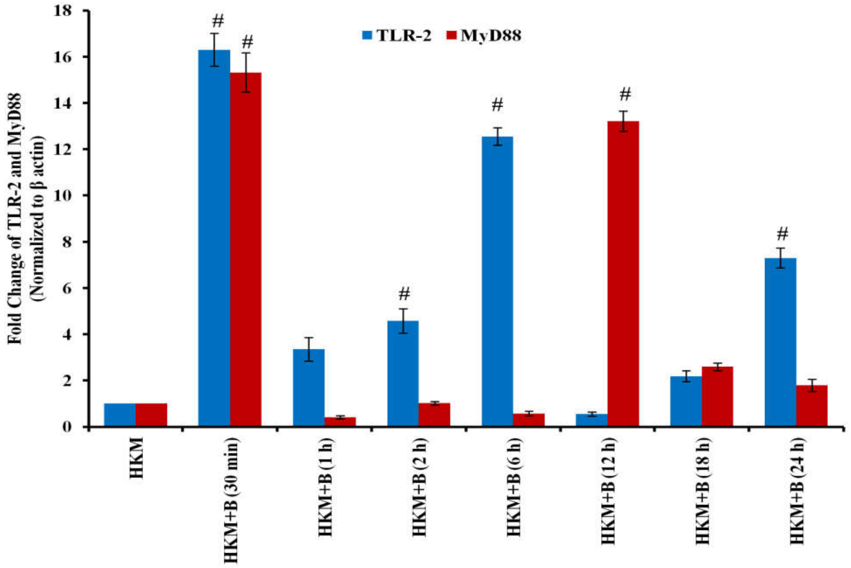

Supplementary Figure 1: *M. smegmatis* activates TLR-2/MyD88 pathway. HKM were infected with *M. smegmatis* and TLR-2 and MyD88 mRNA expression quantified by real-time PCR at indicated time p.i. Vertical bars represent mean ± S.E.M (n=3). $^{\#}P$<0.05 compared to HKM. HKM, uninfected HKM; HKM+B, HKM infected with *M. smegmatis.*

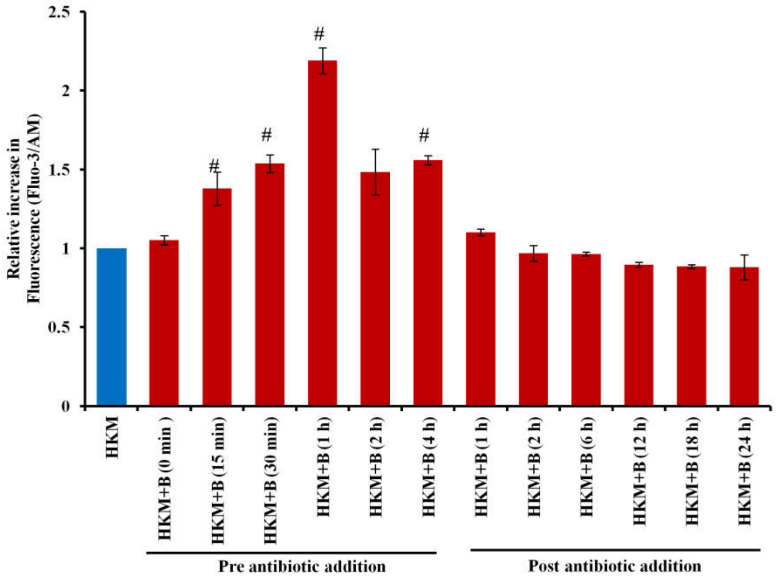

Supplementary Figure 2: ***M. smegmatis*** **elevates $(Ca^{2+})_c$ level.** HKM were infected with *M. smegmatis* and relative changes in $(Ca^{2+})_c$ measured using Fluo3/AM. Vertical bars represent mean ± S.E.M (n=3). $^{\#}P<0.05$ compared to HKM. HKM, uninfected HKM; HKM+B, HKM infected with *M. smegmatis.*

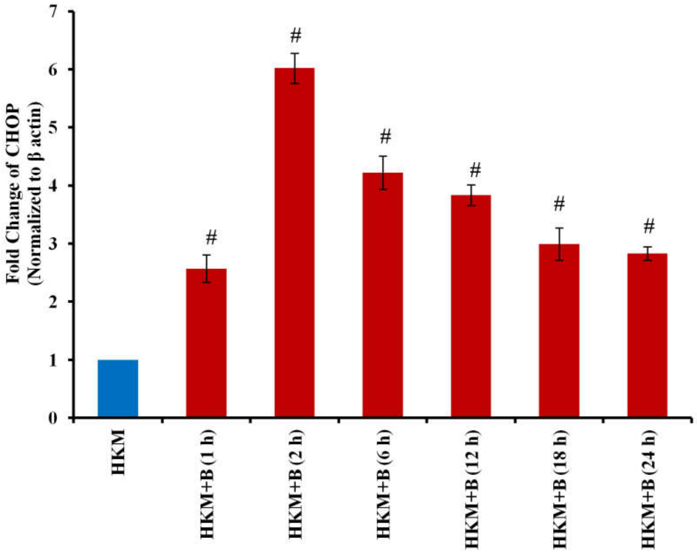

Supplementary Figure 3: ***M. smegmatis*** **induces ER-stress.** HKM were infected with *M. smegmatis* and CHOP mRNA expression quantified by real-time PCR at indicated time p.i. Vertical bars represent mean ± S.E.M (n=3). $^{\#}P<0.05$ compared to HKM. HKM, uninfected HKM; HKM+B, HKM infected with *M. smegmatis.*

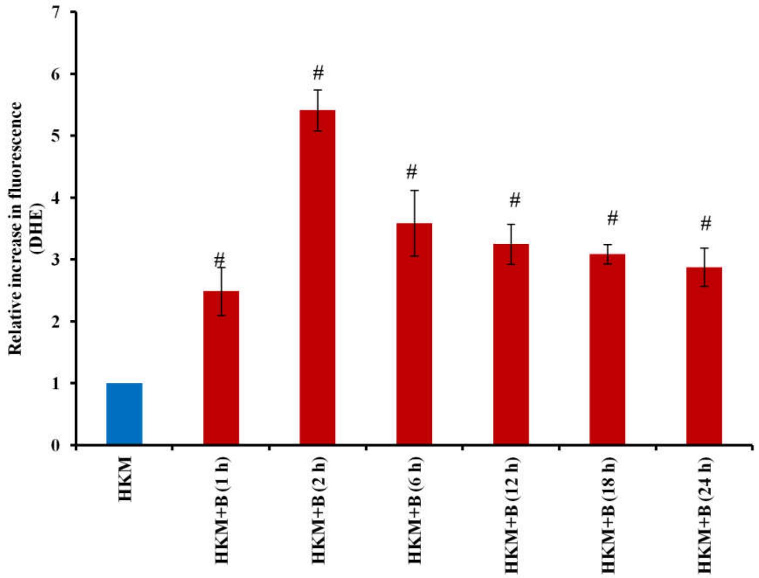

Supplementary Figure 4: *M. smegmatis* triggers O_2^- generation. HKM were infected with *M. smegmatis* and O_2^- generation measured using DHE at indicated time p.i. Vertical bars represent mean ± S.E.M (n=3). $^{\#}P<0.05$ compared to HKM. HKM, uninfected HKM; HKM+B, HKM infected with *M. smegmatis.*

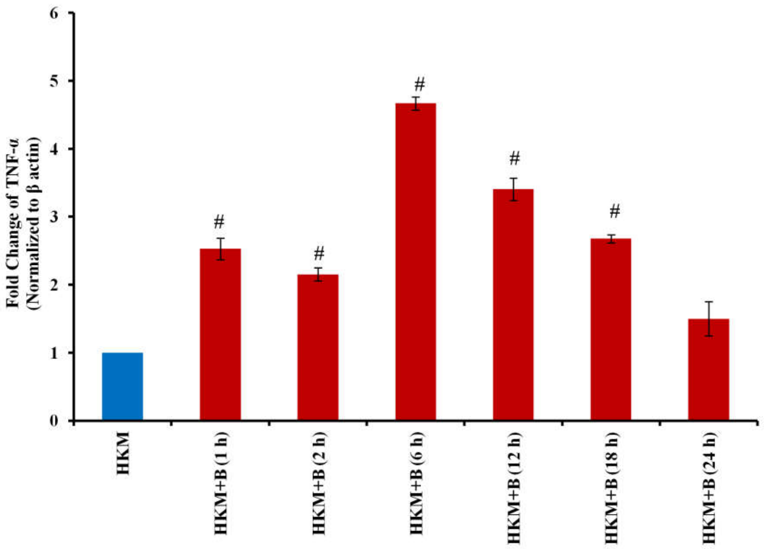

Supplementary Figure 5: *M. smegmatis* induces expression of TNF-α mRNA. HKM were infected with *M. smegmatis* and TNF-α mRNA expression quantified by real-time PCR at indicated time p.i. Vertical bars represent mean ± S.E.M (n=3). $^{\#}P<0.05$ compared to HKM. HKM, uninfected HKM; HKM+B, HKM infected with *M. smegmatis.*

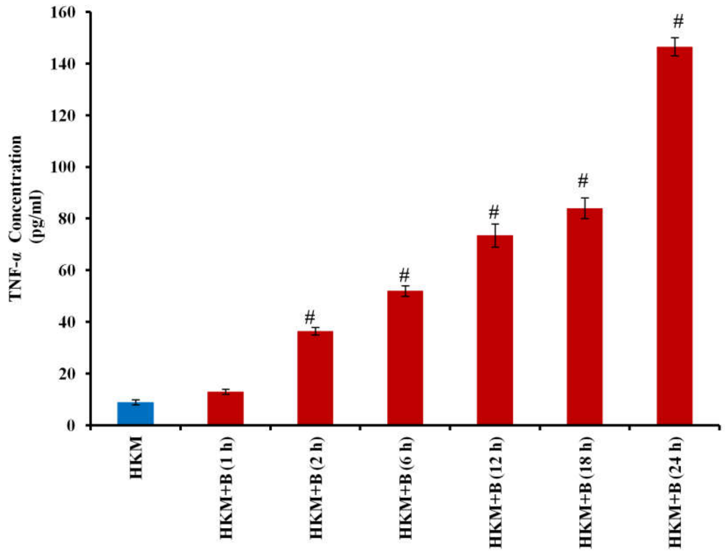

Supplementary Figure 6: *M. smegmatis* induces TNF-α production. HKM were infected with *M. smegmatis* and production of TNF-α protein quantified at indicated time p.i. Vertical bars represent mean ± S.E.M (n=3). #P<0.05 compared to HKM. HKM, uninfected HKM; HKM+B, HKM infected with *M. smegmatis.*

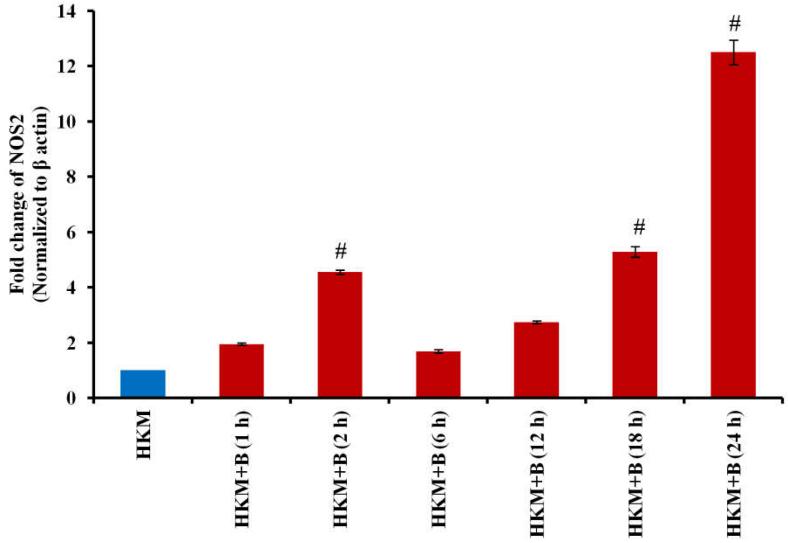

Supplementary Figure 7: *M. smegmatis* induces expression of NOS2 mRNA. HKM were infected with *M. smegmatis* and NOS2 mRNA expression quantified by real-time PCR at indicated time p.i. Vertical bars represent mean ± S.E.M (n=3). #P<0.05 compared to HKM. HKM, uninfected HKM; HKM+B, HKM infected with *M. smegmatis.*

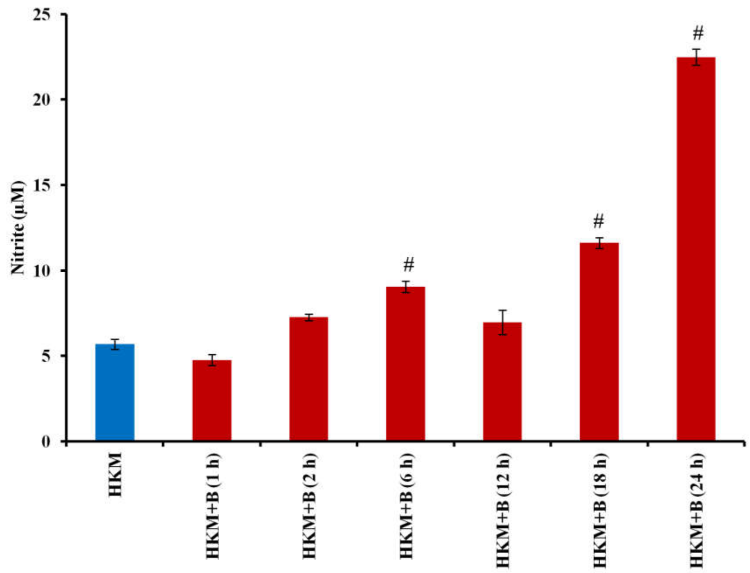

Supplementary Figure 8: *M. smegmatis* induces the production of NO. HKM were infected with *M. smegmatis* and NO production measured at indicated time p.i. Vertical bars represent mean ± S.E.M (n=3). #P<0.05 compared to HKM. HKM, uninfected HKM; HKM+B, HKM infected with *M. smegmatis.*

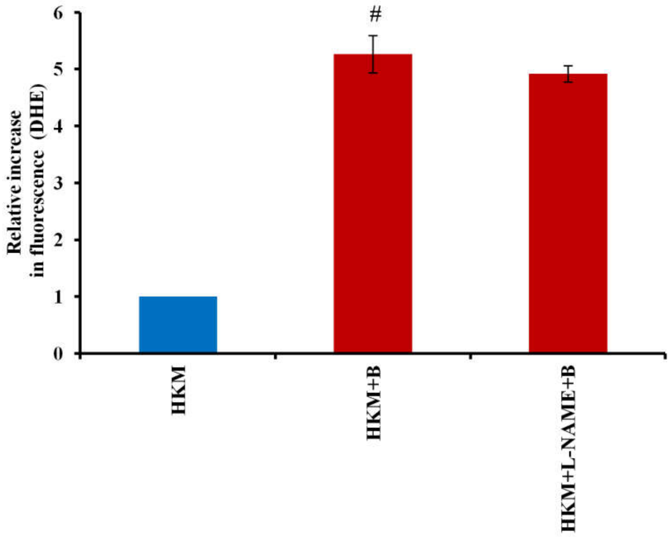

Supplementary Figure 9: **NO has no role in O_2^- generation:** HKM were pre-treated with or without L-NAME prior to infection with *M. smegmatis* and O_2^- generation measured at 2 h p.i. Vertical bars represent mean ± S.E.M (n=3). #P<0.05 compared to HKM. HKM, uninfected HKM; HKM+B, HKM infected with *M. smegmatis,* HKM+L-NAME+B, HKM pre-treated with L-NAME and infected with *M. smegmatis.*

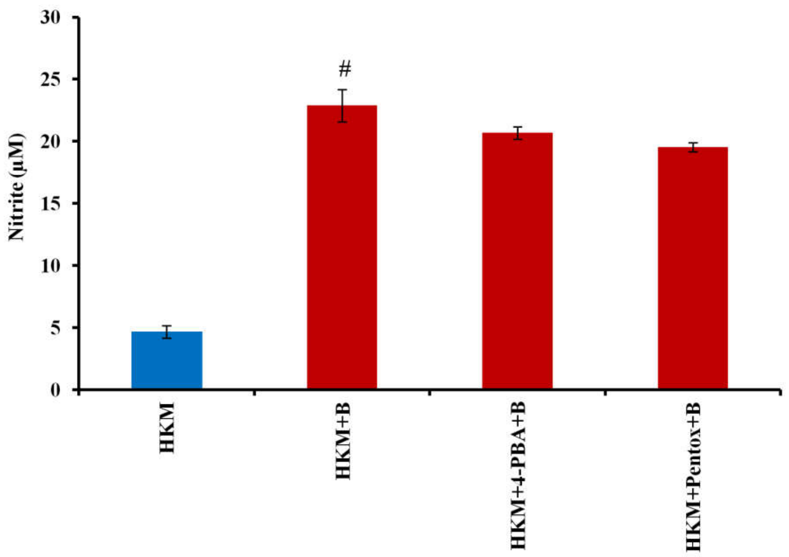

Supplementary Figure 10: ER-stress and TNF-α do not influence NO production. HKM were pre-treated with or without indicated inhibitors prior to infection with *M. smegmatis* and production of NO measured at 24 h p.i. Vertical bars represent mean ± S.E.M (n=3). $^{\#}P<0.05$ compared to HKM. HKM, uninfected HKM; HKM+B, HKM infected with *M. smegmatis,* HKM+4-PBA+B, HKM+Pentox+B, HKM pre-treated with 4-PBA, Pentox respectively and infected with *M. smegmatis.*

Gene	Primer sequences
M. smegmatis **primers**	
**16S rDNA**	FP: 5' -TATCCCAAAGTGCAGGGCAG- 3'<br>RP: 5' -GGCGTGCTTAACACATGCAA- 3'
**ESAT-6**	FP: 5' -CACCTCCAACGAGCTGAACC- 3'<br>RP: 5' -GCAAACATTCCCGTGACGC- 3'
**CFP-10**	FP: 5' -GCAGGTTCAGGAGTTGAACG- 3'<br>RP: 5' -CAGATGTTCATCGACGACGC- 3'
C. gariepinus **primers**	
**TLR-2**	FP: 5' -GGCCGGCAAGTCTCAGGTTTATG- 3'<br>RP: 5' -CGCCATCAGGTCGCTTTTGTTG- 3'
**MyD88**	FP: 5' -CTGAAGCTGTGCGTGTTTGA- 3'<br>RP: 5' -CACCATCCTCTTGCACCTTCT- 3'
**CHOP**	FP: 5' -GTTGGAGGCGTGGTATGAAG- 3'<br>RP: 5'-GAAACTCCGGCTCTTTCTCG- 3'
**TNF-α**	FP: 5' –TCTCAGGTCAATACAACCCGC- 3'<br>RP: 5'-GAGGCCTTTGCGGAAAATCTTG- 3'
**NOS2**	FP: 5'-GACCATCACAGACCACCACA- 3'<br>RP: 5'-GACATAGGAGGTACCAGCCAA- 3'
**β-actin**	FP: 5' –CGAGCAGGAGATGGGAACC- 3'<br>RP: 5'-CAACGGAAACGCTCATTGC- 3'

Supplementary Table 1: List of real-time PCR primers.

Gene	siRNA sequences
**TLR-2**	Sense: 5’-GUACUUGGACUUGAGCCAA- 3’<br>Anti-sense: 5’-UUGGCUCAAGUCCAAGUAC- 3’
**CHOP**	Sense: 5’-AUGAAGACUUGCAAGAUAU- 3’<br>Anti-sense: 5’-AUAUCUUGCAAGUCUUCAU- 3’
**TNF-α**	Sense: 5´-GCAAAGGCCUCUACUUCGU- 3´<br>Anti-sense: 5´-ACGAAGUAGAGGCCUUUGC- 3´
**NOS2**	Sense: 5´-CGCUACAACAUUCUUGAGA- 3´<br>Anti-sense: 5´-UCUCAAGAAUGUUGUAGCG- 3

Supplementary Table 2: List of siRNAs.
